# Filling the Gap to Address Vaccine Hesitancy in Europe

**DOI:** 10.3389/phrs.2025.1608208

**Published:** 2025-01-24

**Authors:** Tiago Correia, Anabela da Conceição Pereira, Henrique Barros, Nadav Davidovitch, Lore Leighton, Alison Katherine McCallum, Paula Meireles, Judith E. Mueller, Robert Otok, Anna Odone, Alena Petrakova, Roman Prymula, Walter Ricciardi, Silvia Gabriela Scintee, Carlo Signorelli

**Affiliations:** ^1^ Global Health and Tropical Medicine, Associate Laboratory in Translation and Innovation Towards Global Health and World Health Organization (WHO) Collaborating Center for Health Workforce Policies and Planning, Institute of Hygiene and Tropical Medicine, Universidade Nova de Lisboa, Lisboa, Portugal; ^2^ Global Health and Tropical Medicine, Associate Laboratory in Translation and Innovation Towards Global Health, Institute of Hygiene and Tropical Medicine, New University of Lisbon, Lisbon, Portugal; ^3^ Centre for Research and Studies in Sociology, Iscte—University Institute of Lisbon (Iscte-IUL), University Institute of Lisbon (ISCTE), Lisbon, Portugal; ^4^ EPIUnit ITR, Institute of Public Health of the University Porto, University of Porto, Porto, Portugal; ^5^ School of Public Health, Faculty of Health Sciences, Ben-Gurion University of the Negev, Beer-Sheva, Israel; ^6^ Association of Schools of Public Health in the European Region (ASPHER), Brussels, Belgium; ^7^ Centre for Population Health Sciences, College of Medicine and Veterinary Medicine, Usher Institute, University of Edinburgh, Edinburgh, United Kingdom; ^8^ Intitut Pasteur, Emerging Disease Epidemiology Unit, Université Paris Cité, Paris, France; ^9^ Université Rennes, Ecole des Hautes Études en Santé Publique (EHESP), CNRS, Inserm, Arènes - UMR 6051, RSMS (Recherche sur les Services et Management en Santé)-U 1309, Rennes, France; ^10^ Unit of Forensic Medicine and Forensic Sciences, Department of Public Health, Experimental and Forensic Medicine, University of Pavia, Pavia, Italy; ^11^ Medical Direction, Fondazione IRCCS Policlinico San Matteo, San Matteo Hospital Foundation (IRCCS), Pavia, Italy; ^12^ Faculty of Health Sciences, Palacký University Olomouc, Olomouc, Czechia; ^13^ Department of Preventive Medicine, Faculty of Medicine in Hradec Králové, Charles University, Hradec Kralove, Czechia; ^14^ School of Public Health, Postgraduate Medical School, Prague, Czechia; ^15^ Department of Health Sciences and Public Health, Università Cattolica del Sacro Cuore, Rome, Italy; ^16^ National Institute of Health Services and Management, Bucharest, Romania; ^17^ Faculty of Medicine and Surgery, School of Hygiene and Public Health, University Vita-Salute San Raffaele, Milan, Italy

**Keywords:** vaccine hesitancy, frontline healthcare workers, vulnerable populations, vaccine interventions, Vax-action

## Introduction

Vaccine hesitancy (VH) is a state of indecision regarding vaccination, marked by doubts despite vaccine availability [1].

Its relationship with vaccine uptake has been widely debated, though the cause-effect relationship remains unclear. Nevertheless, VH likely threatens the control of vaccine-preventable diseases, such as measles and hepatitis B, imposing unnecessary burdens on health systems.

Inappropriate access to information, inadequate vaccine offers, and administration have been identified as primary contributors to reluctance and doubts surrounding vaccine uptake. However, social and individual factors related to knowledge and attitudes further prolong this psychological state of indecision, leading to delayed vaccinations and even refusal. Despite extensive efforts to promote vaccination across European countries, VH persists, affecting vaccine uptake across various demographic groups and settings. Hesitancy varies by country, type of vaccine (whether recently approved or longstanding), and target populations, including children, vulnerable groups, or the general population.

Assessing the frequency, determinants, and impact of vaccine hesitancy presents a significant global and regional challenge [2]. Variations in definitions, data gaps regarding vaccine acceptance, and population coverage hinder precise evaluation. These discrepancies make it more difficult to develop targeted interventions and allocate resources to promote vaccination in a targeted manner and thus effectively improve vaccine acceptance on a large scale.

## Better Evidence and Cooperation to Tackle Vaccine Hesitancy

VH has garnered global attention, particularly in Europe, the US, and Africa. As a result, multiple reports and studies have been published, including the determinants and consequences of vaccine hesitancy.

While many studies and reports focus on VH determinants and consequences, emphasis is now shifting toward improved strategies and recommendations [3], particularly through collaborations like EU-funded research consortia [4, 5].

Healthcare workers play a crucial role in countering VH. Training in communication, managing misinformation, and enhancing self-assurance are key strategies for interacting with hesitant individuals, including parents seeking advice on children’s vaccination [6], and underserved communities, such as migrants [7], ethnically diverse [8], and prisoners [9].

A second focus is on the general population, aiming to combat misinformation and build trust in healthcare and science.

## The Gap to Fill

Despite increasing research on VH, there’s a limited understanding of how to address it effectively. Key questions remain: Is VH a symptom of systemic inequities, or is it an inherent phenomenon in global societies? Should interventions target public health systems or individual decision-making? Understanding the complexities of VH within local and global contexts is essential. Europe lacks a unified framework for addressing VH. While some efforts show promise, a cohesive strategy for interventions remains absent.

It is also essential to address the ethical dimension, to be able to deal with traditional bioethical individual questions, but also focus on the dilemmas resulting from the unknowable statistical lives central in public health reasoning [10].

The gaps in evidence on interventions to address vaccine hesitancy can be summarized as follows:

• There is a need to map interventions to improve individual, communities and societal attitudes towards vaccination and to increase vaccine acceptance, intention, and uptake. Analyzing the performance of prior interventions will help to redefine future interventions and assess their efficacy in the EU and other countries.
• It is essential to identify the problems currently faced by implementing interventions in the field by considering the specificities of target countries and regions, their unique attributes, determinants, and the challenges they encounter.
• Interventions need to be designed based on research findings and previous activities, optimizing resources and actions, including current regular immunization procedures and follow-up vaccination intentions.
• Pilot activities need to be evaluated and implemented. This includes initiatives with the expected highest likelihood of success, such as training courses for frontline health workers and public awareness campaigns to combat vaccine hesitancy. Access to real time data is crucial.

In summary, there is an overall need to create a strong research-grounded sustainability strategy for ongoing implementations and interventions to tackle vaccine hesitancy, and toolkits and suggestions for scaling up in additional EU countries and beyond.

## A Call to Move Forward and an Introduction to Vax-Action

Based on this needs assessment, we call on the scientific community and science funders to strengthen collaborative efforts to monitor, evaluate, and scale up interventions designed to address vaccine hesitancy. The authors propose contributing to this goal by leading a European consortium entitled “VAX-Action, tackling effectively vaccine hesitancy in Europe.” This 30-month project was launched in December 2023 and is co-funded by the European Union’s EU4H program (Grant Agreement No 101133273).

VAX-ACTION aims to make recommendations for action that support decision-makers to address vaccine hesitancy. Five EU countries – Portugal, France, Italy, Romania, and Czechia – will conduct the project, taking advantage of their unique features, including size, vaccination coverage, healthcare system, immunization programs (vaccination settings, involvement of family doctors, pharmacies, hospitals), and the population’s trust in vaccines.

Since there is currently a paucity of evidence regarding the success of previous and ongoing interventions, VAX-ACTION aims to fill the urgent need to support the identification and implementation of key public health findings resulting from the surge in initiatives in the field of vaccination and vaccine hesitancy in Europe, notably:

• To ground in many of those initiatives that have already demonstrated encouraging results, whether for COVID-19 or routine vaccination, particularly catch-up vaccination that was neglected during the peak of the COVID-19 pandemic;
• To contribute to improving vaccine uptake and immunization rates by evaluating best practices and implementing strategies and activities that go beyond European regions and COVID-19 cases;
• To enhance vaccination intervention strategies and planning, advancing personnel planning and organization, and administering vaccinations as intended by countries and the current needs (e.g., new delivery models such as mobile units, preparing for future outbreaks);
• To evaluate whether findings may be used for future public health emergencies requiring mass vaccination or extensive routine immunization programs.

Vax-Action aims to implement a framework that can and should be used more broadly to address interventions in vaccine hesitancy, namely:

1. Action plans to address vaccine hesitancy and improve the understanding of and approaches to:
  • Health literacy, particularly gaps in knowledge and exposure to misinformation;
  • Access, socioeconomic, practical problems, and concerns relating to prior experience;
  • Developing a shared understanding of the importance and benefits of immunization;
  • Positive experiences of immunization;
  • Concerns about risks and over-medicalization;
  • Trust towards the healthcare system, respecting cultural and personal beliefs.
2. Training for health professionals and officials in communication behaviors helping them to anticipate and address potential vaccine hesitancy.
3. Regular evaluation and revision of applied frameworks/models as new evidence emerges.
4. Update experts in healthcare journalism and social media communications to minimize the risk of compromising the public’s right to timely and accurate information from official sources and traditional and digital media.
5. Better engagement with communities to co-design programs, ensuring an active and robust two-way communication mechanism.
6. Rights-based approach to target vaccination support to underserved communities.
7. Further research to evaluate the overall effectiveness of the actual model and ease of implementation as part of an outbreak/pandemic response.
8. Develop existing support mechanisms, such as the WHO immunization strategy process, focusing on global tools and frameworks concerning the development of national cost-effective immunization campaigns.
9. Continue improving existing cross-border, cross-regional, and sub-regional collaboration platforms on information sharing and immunization strategies, learning from past successes and failures.

## References

[1] Bussink-Voorend D Hautvast JLA Vandeberg L Visser O Hulscher MEJL. A Systematic Literature Review to Clarify the Concept of Vaccine Hesitancy. Nat Hum Behav (2022) 6:1634–48. 10.1038/s41562-022-01431-6 35995837

[2] Signorelli C Odone A Ricciardi W Lorenzin B. The Social Responsibility of Public Health: Italy’s Lesson on Vaccine Hesitancy. Eur J Public Health (2019) 29(6):1003–4. 10.1093/eurpub/ckz135 31808818

[3] Sweileh WM. Bibliometric Analysis of Global Scientific Literature on Vaccine Hesitancy in Peer-Reviewed Journals (1990–2019). BMC Public Health (2020) 20(1):1252. 10.1186/s12889-020-09368-z 32807154 PMC7433157

[4] European Centre for Disease Prevention and Control. Vaccine Acceptance Curriculum. Promoting Vaccination Acceptance and Uptake – Communication Strategies for Frontline Health Workers (2022). Available from: https://www.ecdc.europa.eu (Vaccine Acceptance CurriculumFHWs.pdf) Accessed February, 2024.

[5] Betsch C Schmid P Verger P Lewandowsky S Soveri A Hertwig R A Call for Immediate Action to Increase COVID-19 Vaccination Uptake to Prepare for the Third Pandemic Winter. Nat Commun (2022) 13(1):7511. 10.1038/s41467-022-34995-y 36473855 PMC9726862

[6] VAX-TRUST, EU-Research Project 2021-2024. VAX-TRUST Addressing Vaccine Hesitancy in Europe Protocol (2023). Available from: https://vax-trust.eu/ (Accessed February, 2024).

[7] AcToVax4NAM. Access to Vaccination for Newly Arrived Migrants. EU-Research Project 2021-2024. Why Is Increased Access to Vaccination for Newly Arrived Migrants Important? (2023). Available from: https://www.accesstovaccination4nam.eu/ (Accessed February, 2024).

[8] RIVER-EU. H2020 Research Project to Reduce Inequalities in Vaccine Uptake 2021-2026. Increase MMR and HPV Vaccine Uptake in Underserved Communities, Thereby Boosting Herd Immunity for All Europe (2023). Available from: https://river-eu.org/ (Accessed February, 2024).

[9] The Global Health Network WEPHREN. RISE-vac Reaching the Hard-to-Reach: Increasing Access and Vaccine Uptake Among the Prison Population in Europe (2023). Available from: https://wephren.tghn.org/rise-vac/ (EU-Research Project 2014-2020) (Accessed February, 2024).

[10] Maddow R. Identifiable Lives. AIDS and the Response to Dehumanization. An Ethical Compass. In: Coming of Age in the 21st Century. Yale University Press (2010).

